# Inferring the Frank–Starling Curve From Simultaneous Venous and Arterial Doppler: Measurements From a Wireless, Wearable Ultrasound Patch

**DOI:** 10.3389/fmedt.2021.676995

**Published:** 2021-05-14

**Authors:** Jon-Émile S. Kenny, Igor Barjaktarevic, David C. Mackenzie, Philippe Rola, Korbin Haycock, Andrew M. Eibl, Joseph K. Eibl

**Affiliations:** ^1^Health Sciences North Research Institute, Sudbury, ON, Canada; ^2^Division of Pulmonary and Critical Care, Department of Medicine, David Geffen School of Medicine at University of California at Los Angeles, Los Angeles, CA, United States; ^3^Department of Emergency Medicine, Maine Medical Center, Portland, Maine; ^4^Tufts University School of Medicine, Boston, MA, United States; ^5^Division of Intensive Care, Santa Cabrini Hospital, Montreal, QC, Canada; ^6^Department of Emergency Medicine, Riverside University Health System Medical Center, Moreno Valley, CA, United States; ^7^Northern Ontario School of Medicine, Sudbury, ON, Canada

**Keywords:** frank-starling mechanism, Doppler ultrasound, velocity time integral, corrected flow time, venous doppler signals, passive leg raise, fluid tolerance

## Abstract

The Frank–Starling relationship is a fundamental concept in cardiovascular physiology, relating change in cardiac filling to its output. Historically, this relationship has been measured by physiologists and clinicians using invasive monitoring tools, relating right atrial pressure (*P*_ra_) to stroke volume (SV) because the *P*_ra_-SV slope has therapeutic implications. For example, a critically ill patient with a flattened *P*_ra_-SV slope may have low *P*_ra_ yet fail to increase SV following additional cardiac filling (e.g., intravenous fluids). Provocative maneuvers such as the passive leg raise (PLR) have been proposed to identify these “fluid non-responders”; however, simultaneously measuring cardiac filling and output *via* non-invasive methods like ultrasound is cumbersome during a PLR. In this *Hypothesis and Theory* submission, we suggest that a wearable Doppler ultrasound can infer the *P*_ra_-SV relationship by simultaneously capturing jugular venous and carotid arterial Doppler in real time. We propose that this method would confirm that low cardiac filling may associate with poor response to additional volume. Additionally, simultaneous assessment of venous filling and arterial output could help interpret and compare provocative maneuvers like the PLR because change in cardiac filling can be confirmed. If our hypothesis is confirmed with future investigation, wearable monitors capable of monitoring both variables of the Frank–Starling relation could be helpful in the ICU and other less acute patient settings.

## Introduction

**Figure 1:**
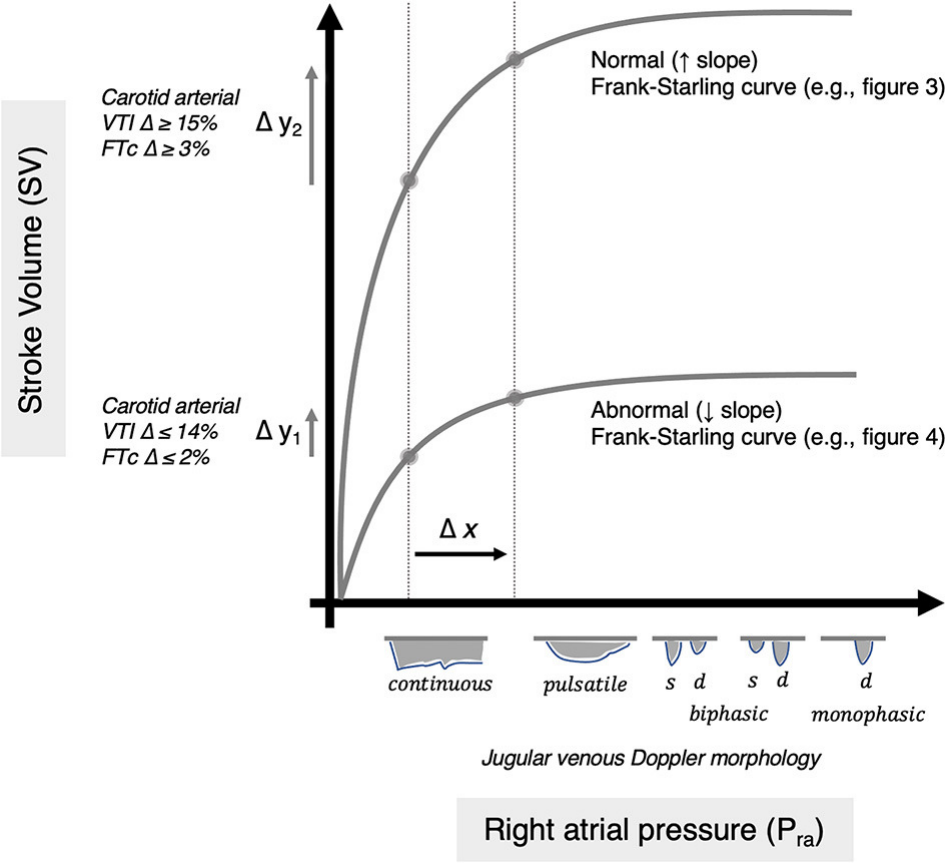
Depiction of normal and abnormal Frank–Starling relationships. The abscissa is *P*_ra_ or right atrial pressure as classically measured (e.g., by a pulmonary artery catheter). The venous Doppler morphology is depicted as a surrogate for *P*_ra_ (2, 3). The ordinate is stroke volume as classically measured (e.g., by a pulmonary artery catheter). The change in carotid arterial Doppler is shown as a surrogate for changing stroke volume (4, 5). Two theoretical curves are depicted with equal change in *P*_ra_, for example from a passive leg raise (arrow Δ*x*). Two potential responses in SV are observed: (1) abnormal, unhealthy (Δ*y*_1_) and (2) normal, healthy (Δ*y*_2_) such that two semiquantitative slopes (Δ*y*/Δ*x*) are inferred.

When receiving more blood from peripheral tissues, cardiac myocytes elongate and contract with greater force—ensuring that the heart ejects what it receives (1); this fundamental attribute is known as the Frank–Starling mechanism. Historically, the Frank–Starling relationship is illustrated with right atrial pressure (*P*_ra_, a surrogate for cardiac filling volume) on the *x*-axis and stroke volume (SV) on the *y*-axis (Figure 1). Clinicians and physiologists have used the slope of the P_ra_-SV relationship to define the adequacy of cardiac function; normally, a small increase in *P*_ra_ leads to a large increase in SV (6).

With acute and chronic disease, however, the slope of the Frank–Starling curve can flatten (7, 8) (Figure 1). This is especially important in critical illness when cardiac function changes rapidly as a consequence of disease and therapy. A diminished *P*_ra_-SV relationship in the intensive care unit (ICU) is particularly important to capture because it may represent a state of “volume unresponsiveness” (9), that is, when SV does not rise in response to augmented cardiac filling volume (e.g., intravenous fluids). In general, roughly 50% of critically ill patients are in a state of “volume unresponsiveness” such that providing intravenous fluids to augment SV is unhelpful (10). Given that excessive, intravenous fluid in the ICU is associated with adverse events (11) and that targeted administration of intravenous fluids improves patient-centered outcomes (9, 12), delivering fluid therapy in the ICU based on functional hemodynamic monitoring (FHM) has become standard-of-care (13–15).

FHM is a physiology-based paradigm that centers intravenous fluid therapy around the slope of the Frank–Starling curve rather than absolute values of cardiac filling pressure or output. Importantly, to define the slope of the curve, the change in SV (*y*-axis) is measured following the change in right atrial pressure (*x*-axis) (Figure 1). Traditionally, clinicians used invasive monitoring tools such as the pulmonary artery catheter—where cardiac filling pressure and output are quantified in response to intravenous fluids. However, in the contemporary ICU, invasive monitors are used less frequently, being replaced with non-invasive methodologies (16). One approach to measure SV is with Doppler ultrasound following increased cardiac filling induced by a passive leg raise (PLR) maneuver (6). Yet, the workflow is cumbersome to perform with a handheld ultrasound, and when done, clinicians typically only measure the change in SV (i.e., *y*-axis) while assuming that the PLR maneuver increases cardiac filling (i.e., the *x*-axis). Indeed, experts in FHM stress that increased cardiac volume has to be confirmed to rise during the PLR (17), though this is rarely done because it necessitates either invasive monitoring or because it is difficult to non-invasively monitor both cardiac inflow and outflow, synchronously.

## The Hypothesis

Given that arterial Doppler ultrasound tracks SV (18, 19) and venous Doppler qualitatively changes in response to increased right atrial pressure (2, 3), we hypothesize that a wireless, wearable Doppler ultrasound simultaneously insonating the common carotid artery and internal jugular vein will demonstrate the Frank–Starling relationship in real time. In this manner, hands-free ultrasound can identify patients with low cardiac filling pressure who are, nevertheless, volume “unresponsive” and also determine if provocative maneuvers like the PLR augment cardiac filling.

## Clinical Considerations and Description of the Wearable Doppler

In the ICU, the relationship between *P*_ra_ and the slope of the Frank–Starling curve is variable, even at low *P*_ra_. As above, this speaks to the high frequency of critically ill patients with reduced *P*_ra_-SV slope. More specifically, 20–40% of patients with a “low” *P*_ra_ (e.g., <5–8 mmHg) are “volume unresponsive” (20, 21). These data have been borne out using non-invasive, ultrasonographic surrogates for the *P*_ra_ such as collapse of the inferior vena cava (IVC). Inspiratory collapse of the IVC is present in ~20–30% of patients who do *not* augment SV in response to intravenous fluids (22).

Importantly, the qualitative morphology of the *venous* Doppler velocity envelope also changes as a function of *P*_ra_. This is demonstrated in multiple veins including the hepatic (23), portal (24), common femoral (25), internal jugular (26), and the superior vena cava (27). Moreover, Iida and Tang have, more recently, observed and illustrated the aforementioned changes in the intrarenal venous Doppler waveform in heart failure patients (2, 3). Given that many ICU patients have a diminished Frank–Starling slope (7, 8) despite having low *P*_ra_ and/or IVC collapse (20–22), we suspect that some ICU patients will have jugular venous Doppler velocimetry consistent with low *P*_ra_, yet not augment SV in response to a PLR. Furthermore, we anticipate that a PLR will modify the internal jugular venous Doppler consistent with rising *P*_ra_–qualitatively tracking the *x*-axis of the Frank–Starling relationship (Figure 1).

Association between changing SV and common carotid *arterial* Doppler in critically ill patients has been demonstrated (18, 19). This is noted for both total flow and corrected systolic flow time as a surrogate for SV and supported by a recent systematic review (28). Nevertheless, there are conflicting data on this topic (29); there are potentially many sources of error that could explain discrepant results including variability in devices used, patient demography, and protocol differences (30, 31). Additionally, as human sampling error affects handheld carotid Doppler measurements (30, 32), it may be that manual manipulation and differences in the number of beats sampled account for conflicting results.

To mitigate some of the aforementioned sources of error, we have deployed a wireless, wearable, continuous wave, 4-MHz Doppler ultrasound patch that generates a broad beam capable of insonating both the common carotid artery and internal jugular vein, simultaneously (33, 34). More specifically, the device (Flosonics Medical, Sudbury, Ontario, Canada) insonates a field ~3 to 4 times the width of a normal carotid artery such that both common carotid arterial and jugular venous spectra are visually and audibly displayed to the clinician *via* the user interface. A recent usability comprising physicians, nurses, and lay users showed that Doppler spectra were quickly and easily obtained by all after a brief training video (34).

The wearable ultrasound adheres to the neck and maintains a constant angle of insonation relative to the carotid and jugular vein even when a patient is made supine during a PLR, so long as neck position does not change during the maneuver (34). Also, the device averages cardiac cycles over many seconds which attenuates handheld sampling variability (30, 31).

The wearable Doppler ultrasound has been studied in healthy volunteers where an excellent association between changing SV and carotid artery Doppler was observed (4, 5). More specifically, we have shown that a clinically significant change in SV (i.e., more than 10%) was captured by a rise in carotid artery velocity time integral (VTI) and FTc of at least 15 and 3–4%, respectively (4, 5).

## Proposed Evaluation Of The Hypothesis

Given the unique ability of the ultrasound patch to simultaneously insonate both the carotid artery and jugular vein, we hypothesize that instantaneous assessment of venous and arterial Doppler during a PLR could intimate the slope of the *P*_ra_-SV relationship. Importantly, the *P*_ra_-SV slope evolves *as a consequence* of the PLR (Figure 1)—it is not that an absolute slope of the curve is calculated before and after the PLR. Accordingly, this approach requires neither the absolute value of *P*_ra_ nor SV but rather demands simply that their change be tracked in real time. In other words, measuring how the common carotid artery Doppler profile *changes* during the PLR infers ΔSV (Δ*y* of the *P*_ra_-SV relationship), while venous Doppler qualitatively tracks the Δ*P*_ra_ (Δ*x* of the *P*_ra_-SV relationship). Therefore, in this hypothesis, we emphasize that using venous and arterial Doppler to infer changes in *P*_ra_ and SV are qualitative and semiquantitative approaches, respectively, to obtain ΔSV/Δ*P*_ra_ induced by PLR. For example, with a normal, steep *P*_ra_-SV relationship, PLR is expected to induce a relatively large ΔSV/Δ*P*_ra_. By contrast, a diminished *P*_ra_-SV slope with low, baseline *P*_ra_ should reveal venous waveform changes consistent with low, but rising, *P*_ra_ and minimal elevation of arterial flow induced by PLR (Figure 1), that is, a comparatively low ΔSV/Δ*P*_ra_.

## Proof-of-Concept Data

**Figure 2:**
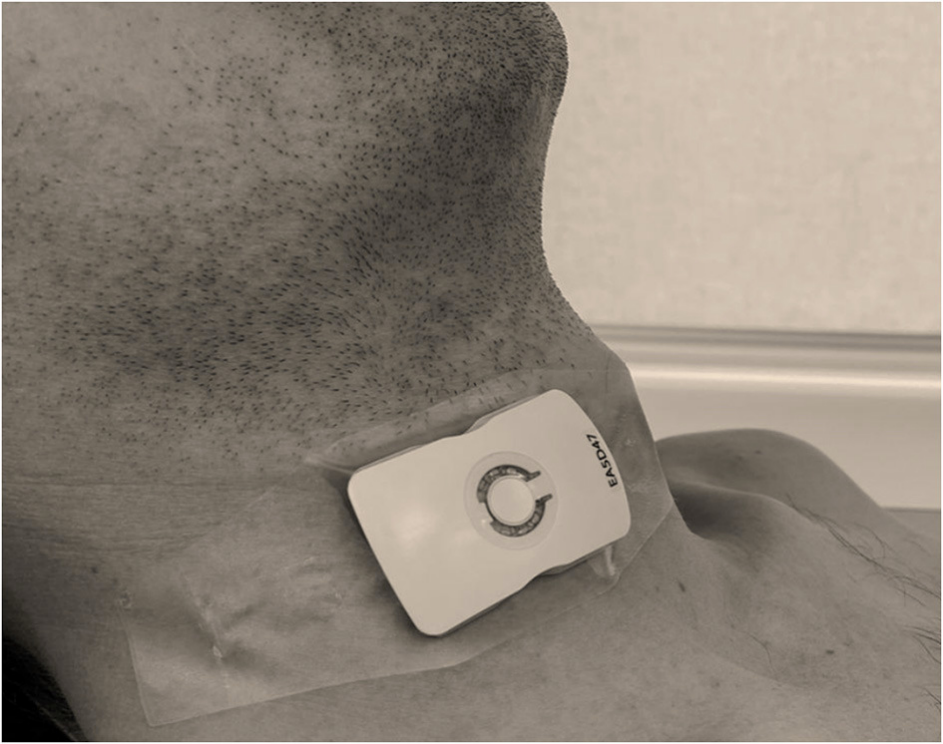
Picture of wireless, wearable Doppler ultrasound.

Herein, we present two cases of simultaneously recorded internal jugular vein and common carotid artery Doppler signals *via* a wireless, wearable ultrasound patch (Figure 2) during PLR. The first is a healthy 25-year-old man also wearing a non-invasive, volume-clamp SV monitor (ClearSight, Edwards Lifesciences, Irvine, CA). The second is a previously healthy, 47-year-old, spontaneously breathing (i.e., not mechanically ventilated) woman admitted to the ICU for septic shock from pneumonia; her SV was simultaneously monitored *via* bioreactance (Cheetah NICOM, Baxter Medical, Deerfield, IL). Both underwent a 1-min baseline period followed by a 3-min PLR.

The SV change recorded by bioreactance was obtained from the automated “*Starling Report*” generated by the NICOM device, in accordance with the manufacturer's recommendation and as utilized for clinical decision-making. The SV change measured by the non-invasive pulse contour device followed the protocol employed by the group who originally proposed the passive leg raise paradigm—as described previously (17, 35). The reason for the different gold standards was dictated by local preference and convenience at the time of assessment; these illustrative cases were chosen because both had similar baseline jugular venous morphology, but, with PLR, had different ΔSV/Δ*P*_ra_ responses per the hypothesis described above and elaborated upon below.

**Figure 3:**
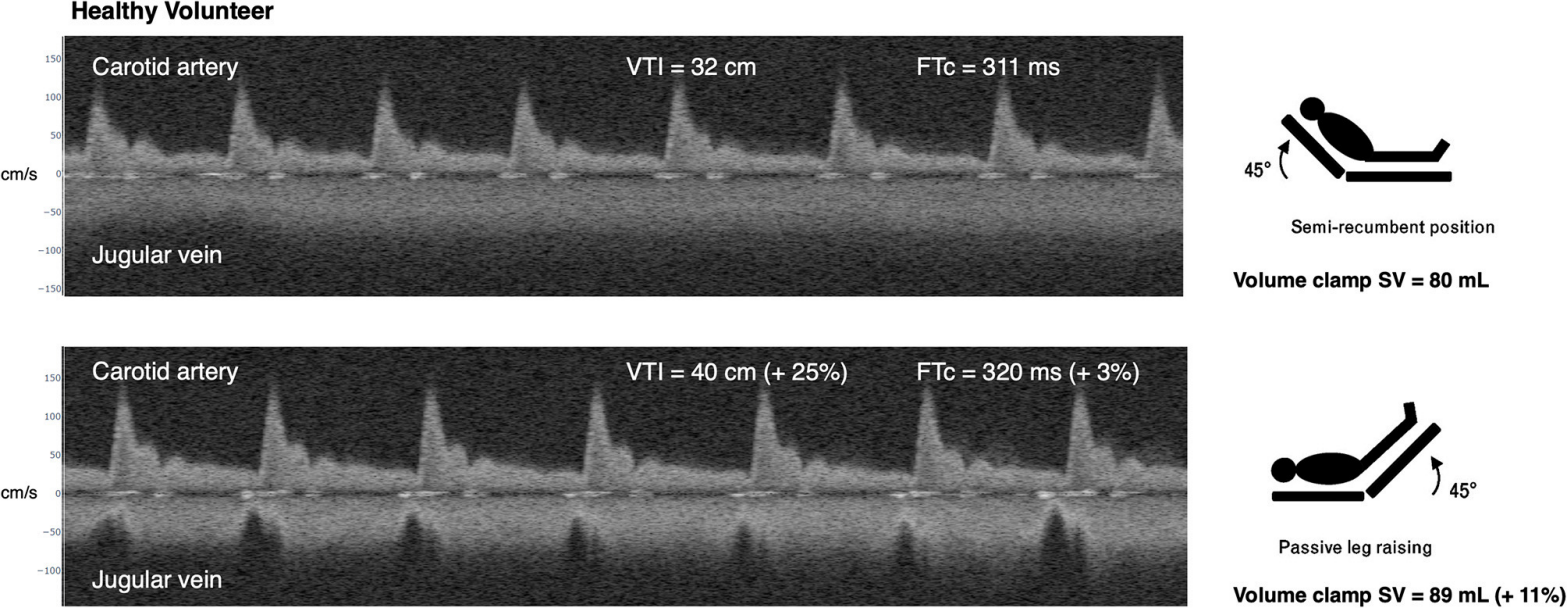
Example of simultaneous carotid arterial and jugular venous Doppler in healthy subject during passive leg raise. At baseline, in semirecumbent, the subject has continuous (low *P*_ra_) jugular venous Doppler morphology. Upon passive leg raise (PLR), the venous morphology transitions to a pulsatile morphology suggesting a rise in *P*_ra._ There is augmentation of carotid arterial Doppler metrics during PLR and SV by non-invasive volume-clamp. Compare this response to the “normal” *P*_ra_-SV relationship in Figure 1. VTI is velocity time integral in centimeters, cm. FTc is corrected flow time in milliseconds, ms. SV is stroke volume in milliliters, ml. cm/s is centimeters per second.

In the healthy subject, the baseline venous Doppler velocity envelope is continuous, high velocity, and minimally undulating. Per the Iida and Tang classification (2, 3), this illustrates low *P*_ra_. More specifically, Iida and colleagues found that continuous, biphasic, and monophasic venous Doppler morphology is related to *P*_ra_ values of 5.4, 9.5, and 14.9 mmHg, respectively (2, 3). The high, continuous venous velocity is likely due to the Bernoulli principle in a partially collapsed, ellipsoid, jugular vein. Upon PLR, the venous waveform becomes somewhat pulsatile intimating a rise in *P*_ra_ (Figure 3). Concomitantly, the increase in carotid arterial VTI and corrected systolic flow time demonstrates clinically significant SV augmentation (4, 5), which was confirmed by the non-invasive volume-clamp SV monitor. Thus, from the wearable Doppler, the subject was inferred to have a relatively large ΔSV/Δ*P*_ra_.

**Figure 4:**
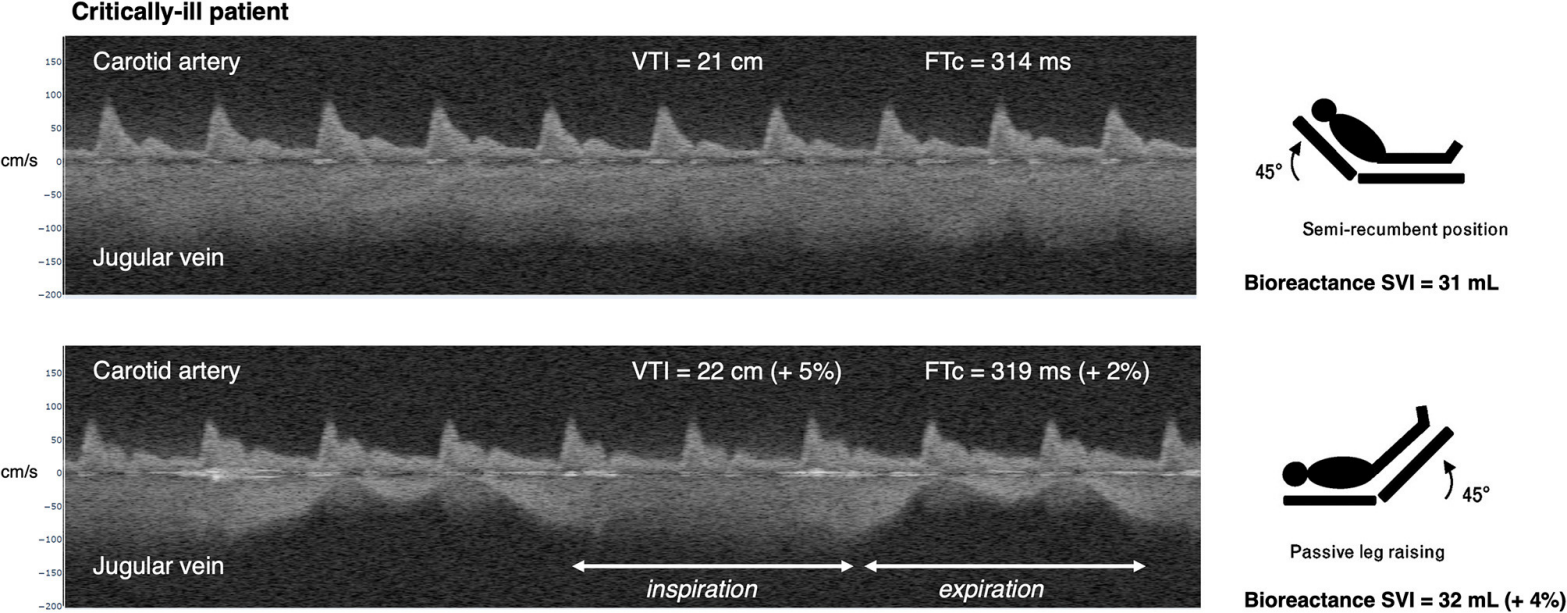
Example of simultaneous carotid arterial and jugular venous Doppler in a patient with septic shock during passive leg raise. At baseline, in semirecumbent, the patient has continuous (low *P*_ra_) jugular venous Doppler morphology. Upon PLR, the venous morphology transitions to a pulsatile waveform on expiration. On inspiration, the velocity is high, suggesting inspiratory collapse of the jugular vein. There is no augmentation of carotid arterial Doppler metrics during PLR. Compare this response to the “abnormal” *P*_ra_-SV relationship in Figure 1. VTI is velocity time integral in centimeters, cm. FTc is corrected flow time in milliseconds, ms. SVI is stroke volume index in milliliters, ml. cm/s is centimeters per second.

In the critically ill patient, baseline jugular vein morphology is also continuous with high velocity consistent with a partially collapsed vein (Figure 4). Upon PLR, the venous morphology varies with respiration. On inspiration, the velocity is high and continuous suggesting partial vein collapse, while on expiration, the velocity falls into a pulsatile morphology consistent with rising *P*_ra_. Nevertheless, the patient has no significant change in carotid arterial VTI (21 to 22 cm, +5%) or corrected systolic flow time (314 to 319 ms, +2%), demonstrating little change in SV as described previously (4, 5). Bioreactance monitoring confirmed that this patient did not increase SV with PLR (+4%). Thus, from the wearable Doppler, the patient was inferred to have a relatively low ΔSV/Δ*P*_ra_.

## Discussion

In this *Hypothesis and Theory* submission, we provide proof-of-concept data suggesting that real-time, simultaneous Doppler ultrasound of the internal jugular vein and common carotid artery illustrates the Frank–Starling law of the heart. If confirmed in future investigations, this hypothesis has a number of clinically important implications. First, assessing the *P*_ra_-SV relationship gives the clinician a fuller picture of cardiac function as compared with absolute hemodynamic variables such as filling pressure, stroke volume, blood pressure, or ejection fraction (6). Because the slope of the *P*_ra_-SV curve is different between patients and within one patient over the arc of an illness, identifying a *P*_ra_ or SV threshold to guide provision or withdrawal of fluid is not possible. More certainty regarding the *P*_ra_-SV slope could prevent unnecessary and potentially harmful intravenous fluids (9, 11). Recent data and meta-analysis support this approach (9, 12). Second, relating arterial-to-venous Doppler as described herein reiterates that low filling pressure—whether assessed by invasive catheter, inspiratory IVC collapse, or venous Doppler morphology—may be observed in a patient who is nevertheless preload intolerant. In other words, this approach reaffirms that low preload may associate with cardiac fluid unresponsiveness. Third, when the PLR is used as a reversible method to increase cardiac filling, jugular velocimetry helps determine if preload truly changes. While assuring that cardiac volume rises during a PLR is recommended by authorities (17), contemporaneously assessing cardiac filling and stroke volume is challenging with bedside ultrasound. Performing repeat PLR maneuvers in succession may not equally challenge the Frank–Starling mechanism should blood redistribution alter the amount of venous return with each maneuver. Accordingly, if the Frank–Starling mechanism is analogized to a “dose–response” curve, then the venous Doppler velocity morphology tells the clinician that different PLR maneuvers have “dosed” the heart equitably. In this manner, determining that the PLR truly modifies cardiac preload may partly explain conflicting data on the reproducibility of the PLR maneuver (36).

This hypothesis appears to indicate that elevated *P*_ra_ (e.g., a biphasic or monophasic venous Doppler) is universally associated with “volume unresponsiveness”; that is, an elevated *P*_ra_ invariably lands upon the flat portion of the Frank–Starling relationship. While rising *P*_ra_ increases the probability of volume unresponsiveness (20), there may be clinical situations where administering intravenous fluid is beneficial in the context of elevated *P*_ra_. In a recent analysis of septic shock patients, intravenous fluid acted as an inotrope and improved the slope of the Frank–Starling curve (37). Nearly all fluid-naive patients increased cardiac index following intravenous fluids with baseline *P*_ra_ of roughly 8 mmHg. This value of *P*_ra_ is expected to demonstrate a continuous or biphasic pattern of venous Doppler based on the Iida classification (3). Nevertheless, fluids augmented SV with little change in the *P*_ra_ in fluid-naive septic shock patients. Accordingly, patients with biphasic venous Doppler at baseline *may* benefit physiologically from fluids in the correct clinical context. In theory, PLR could help define this without the risk of unnecessary fluids.

In situations where excessive external pressure retards venous return to the right heart (e.g., tension pneumothorax, pericardial tamponade, dynamic hyperinflation), *P*_ra_ rises relative to atmospheric pressure; however, *P*_ra_ *transmural* pressure falls and the right heart shrinks in size. As the change in the venous Doppler waveform is thought partly due to the right atrium stretched to its elastic limits (i.e., elevated transmural *P*_ra_), the change in venous Doppler morphology secondary to external compression may not follow the pattern put forth in this hypothesis. Thus, patients with these pathologies require investigation before applying the physiological framework described above.

The effect of respiration on jugular venous Doppler morphology is also noteworthy. Presumably, when right atrial or central venous pressure is low, the vein is partially collapsed or ellipsoid, resulting in a relatively high-velocity, amorphous trace because of the Bernoulli principle. Nevertheless, with excessive inspiratory effort (+/– mechanical ventilation), *P*_ra_ can be sucked below atmospheric pressure and collapse the vein, leading to high jugular velocity despite a truly high *P*_ra_. This is likely observed in the critically ill patient (Figure 4). Accordingly, the morphology of the venous trace might best approximate *P*_ra_ at *end-expiration*—when intrathoracic pressure is closest to atmospheric pressure and so intravascular pressure best approximates its transmural pressure. Indeed, end-expiration is when invasively measured *P*_ra_ should be obtained (20). Nevertheless, in patients with extreme respiratory distress, especially those also making active *expiratory* efforts, inferring qualitative change in *P*_ra_ from venous Doppler may be invalid. If the patient is using accessory muscles of inspiration and contracting abdominal muscles on expiration, then respiratory effort is likely too great to interpret venous Doppler morphology. Notwithstanding, when a patient is fully adapted with a mechanical ventilator (i.e., making no respiratory effort), observing the jugular venous morphology at end-expiration remains most appropriate. Additionally, the change in the jugular venous morphology during a standardized, ventilator-delivered breath may divulge supplemental hemodynamic data. Given that passive, inspiratory distention of the jugular vein corresponds with a steep Frank–Starling curve (38), the change from a continuous, high velocity to a lower, potentially pulsatile velocity during a passive, mechanical breath suggests a steep *P*_ra_-SV relationship which could be confirmed with a PLR.

Finally, while resuscitation in the ICU has focused, historically, on forward, arterial flow, there is growing attention to either avoiding over-resuscitation or the concept of timely *de-resuscitation*. Sepsis studies have revealed an association between poorer outcomes and elevated *P*_ra_ (39), Doppler flow abnormalities have been correlated with organ dysfunction (24), and certain groups are advocating de-resuscitation strategies such as the ROSE approach (40). Notably, following successful hemodialysis, carotid Doppler-corrected flow time rises with PLR (41) suggesting a rising *P*_ra_-SV slope. Additionally, loss of pulsatile venous morphology is observed with successful blood volume removal in the ICU (42). With this perspective, accurate, multimodal data triangulate the patient's physiological state and help the clinician select and titrate therapy accordingly. Thus, in the clinical context of volume overload, a rising *P*_ra_-SV slope with de-resuscitation therapy supports the benefit of diuresis and may detect the hemodynamic limits of this phase as well.

## Conclusion

In this *Hypothesis and Theory*, we describe the physiological rationale and provide early proof-of-concept, feasibility data suggesting that simultaneous venous and arterial Doppler should be used to infer the slope of the Frank–Starling curve. We make this proposal because clinicians often approach functional hemodynamic monitoring with an implicit understanding of the *P*_ra_-SV relationship but focus their effort on capturing the change in stroke volume while ignoring the *P*_ra_. Furthermore, it is sometimes assumed that low *P*_ra_ equates with a steep Frank–Starling relationship and that provocative maneuvers like the PLR invariably raise cardiac filling. We hypothesize that simultaneous jugular venous and carotid arterial Doppler assessment extrapolates the slope of the *P*_ra_-SV relationship. Accordingly, assessing venous filling and arterial output, in tandem, may prevent harmful fluids when *P*_ra_ is low and when a patient is, nevertheless, volume unresponsive. Also, evaluating the venous waveform ensures that provocative maneuvers like the PLR truly raise cardiac filling volume as anticipated. Non-invasive evaluation of the Frank–Starling slope is important given that it changes rapidly in the ICU as a function of disease and therapy. Finally, should the aforementioned hypotheses prove correct with future investigation, wearable ultrasound monitors capable of assessing the *P*_ra_-SV relationship may be clinically helpful beyond the ICU, for example in dialysis units, the general medical floor, and in the outpatient setting.

## Data Availability Statement

The raw data supporting the conclusions of this article will be made available by the authors, without undue reservation.

## Author Contributions

J-ÉK: conception, analysis, interpretation, and drafting. IB, DM, PR, and KH: analysis, interpretation, and critical revisions. AE and JE: data capture, analysis, interpretation, and critical revisions. All authors contributed to the article and approved the submitted version.

## Conflict of Interest

J-ÉK, JE, and AE are working with Flosonics, a start-up developing a commercial version of the ultrasound patch. IB has received grants and consulting fees for GE Healthcare. The remaining authors declare that the research was conducted in the absence of any commercial or financial relationships that could be construed as a potential conflict of interest.
